# Retinal vessel tortuosity as a prognostic marker for disease severity in Fabry disease

**DOI:** 10.1186/s13023-021-02080-0

**Published:** 2021-11-20

**Authors:** Yevgeniya Atiskova, Jan Wildner, Martin Stephan Spitzer, Charlotte Aries, Nicole Muschol, Simon Dulz

**Affiliations:** 1 Department of Ophthalmology, University Medical Center Hamburg-Eppendorf, Martinistr. 52, 20246 Hamburg, Germany; 2 Department of Pediatrics, University Medical Center Hamburg-Eppendorf, Hamburg, Germany

**Keywords:** Fabry disease, Vascular tortuosity, Retina, OCT-A, MONA REVA

## Abstract

**Purpose:**

The aim of this case control study was to evaluate the prognostic value of automatically quantified retinal vessel tortuosity from fundus images and vessel density from OCT-A in Fabry disease and to evaluate the correlation of these with systemic disease parameters.

**Methods:**

Automatically quantified perimacular retinal vessel tortuosity (MONA REVA software), acquired by fundus imaging, and perifoveal retinal vessel density, acquired by optic coherence tomography angiography (OCT-A) were compared between 26 FD patients and 26 controls. Gender and FD phenotype were analyzed to the obtained retinovascular data and correlated to the Mainz severity score index (MSSI) and plasma lyso-Gb3.

**Results:**

Automatically quantified retinal vessel tortuosity indices of FD patients were significantly lower, reflecting an increased vessel tortuosity, compared to controls (*p* = 0.008). Males with a classical phenotype showed significantly lower retinal vessel tortuosity indices compared to males with an oligosymptomatic phenotype and females with a classical or oligosymptomatic phenotype (*p* < 0.001). The retinal vessel tortuosity index correlated significantly with systemic disease severity parameters [global MSSI (r = − 0.5; *p* < 0.01), cardiovascular MSSI (r = − 0.5; *p* < 0.01), lyso-Gb3 (r = − 0.6; *p* < 0.01)].

**Conclusion:**

We advocate fundus imaging based automatically quantified retinal vessel tortuosity index over OCT-A imaging as it is a quick, non-invasive, easily assessable, objective and reproducible marker.

## Introduction

Fabry disease (FD) is a rare X-chromosomal inherited lysosomal storage disorder affecting the glycosphingolipid metabolism. The prevalence is estimated between 1/40.000 and 1/117.000 [1, 2]. Pathogenic variants of the α-Galactosidase A (*GLA*) gene located at chromosome Xq22 result in decreased activity of the lysosomal enzyme GLA [2, 3]. Glycosphingolipid accumulation in lysosomes of multiple tissues results in non-specific symptoms such as acroparesthesia, cutaneous angiokeratomas, hypoacusia, hypohidrosis, heat/cold and exercise intolerance as well as gastrointestinal pain and motility issues. Severe illness and even death can occur due to progressive renal failure, cerebrovascular events such as transient ischemic attacks (TIA) or stroke or cardiac disease including hypertrophic cardiomyopathy, arrhythmia, heart failure or myocardial infarction [2, 4, 5]. Males with the classic phenotype of the disease develop disease signs in the first decade of life with multi-organ involvement [2, 6], while female heterozygous carrier tend to have a considerably slower disease progression [7]. To assess disease severity and progression, a clinical grading scale the Mainz Severity Score Index as well as serum lyso-Gb3 levels are routinely used in clinical practice [8, 9].

Approved therapeutic options for the treatment of FD are intravenously applied recombinant enzyme replacement therapy (ERT) with agalsidase alfa or agalsidase beta [4, 10] and for specific *GLA* mutations an oral chaperon therapy with migalastat is available [11]. An early diagnosis of FD leads to early access to disease-specific follow-up programs and treatment protocols affecting the prognosis significantly [10].

Ocular signs of FD such as cornea verticillata, subcapsular cataract formation and vessel tortuosity of the conjunctival and retinal vessels can be observed prior or parallel to systemic symptoms and can contribute to finding the diagnosis [12, 13].

Furthermore, we recently reported on intraretinal hyperreflective foci in FD patients identified by optical coherence tomography (OCT) imaging [14]. A positive correlation of these intraretinal hyperreflective foci with the prognostic serological marker lyso-Gb3 was detected, suggesting a possible prognostic value of retinal parameters for the classification of systemic disease severity. We also analyzed retinal vessel tortuosity of FD patients in this former study, which was remarkably increased compared to control persons and correlated significantly with lyso-Gb3 levels [14]. Retinal vessel tortuosity in FD is known to be caused by intracytoplasmic storage of ceramide within endothelial cells [13, 15] and adaptive optics in FD patients has used to outline the perivascular alterations including intracellular sphingolipid storage in the retinal vasculature [16]. As a consequence, the vessel wall resistance to hydrostatic pressure decreases and vascular tortuosity in both arterial and venous vessels develops [17]. Interestingly, Sodi et al. also analyzed retinal vessel tortuosity in FD patients semi-automatically and showed significantly higher values in FD patients compared to a control group [18, 19]. Yet, associations of retinal vessel tortuosity with systemic disease severity were either not found or not analyzed in these studies [18, 19]. San Roman et al. described a significant correlation between semi-automatically assessed retinal vessel tortuosity and systemic severity measured by the global MSSI, renal MSSI, and neurological MSSI despite the small cohort of ten patients [20]. Therefore, retinal vessel tortuosity seems to represent an objective and reproducible tool, which might play a role as a non-invasive diagnostic and prognostic marker in FD, yet the shortcomings of complex post-imaging analysis hindered the daily clinical application.

Retinal vessels of FD patients were also investigated using OCT Angiography (OCT-A), which showed vascular abnormalities as enlarged foveal avascular zones and increased macular vessel density scores in the superficial vascular plexus in FD patients compared to healthy controls in the study of Minnella et al. [21]. On the contrary, Cakmak et al. reported significantly lower macular vessel densities in the superficial vascular plexus in FD patients compared to a control group [22]. The correlation with systemic parameters was not tested in either study. Dogan et al. revealed, that the retinal vessel density in the deep vascular plexus was decreased in FD patients compared to controls, without significant difference in the superficial vascular plexus [23], suggesting inhomogeneous results in the evaluation of vessel characteristics with the aid of OCT-A.

The aim of the presented study was to evaluate different non-invasive methods to quantify retinovascular characteristics of FD patients. We analyzed the perifoveal retinal vessel density of FD patients using OCT-A and applied a commercially available software (MONA REVA software) to assess retinal vessel tortuosity on acquired fundus images. Both methods were analyzed with respect to correlations with established systemic disease parameters to determine a quick, non-invasive, easily assessable, objective and reproducible ophthalmological tool with a possible prognostic value and possible use in clinical routine.

## Methods

The study was performed in accordance with the Declaration of Helsinki and approved by the medical ethics committee of the Ärztekammer Hamburg, Germany. Informed consent was obtained from every individual. All FD patients were recruited in line with the yearly routine ophthalmological exam at the University Medical Center Hamburg-Eppendorf, Germany. Twenty-six patients with genetically confirmed FD and 26 age-matched healthy control persons, who were recruited among healthy coworkers at the University of Hamburg-Eppendorf, were included in the prospective observational, cross sectional, comparative study. Any ophthalmological comorbidities were regarded as exclusion criteria.

### Ophthalmological examination

Comprehensive ophthalmological investigation was performed on all participants, including medical history, best-corrected visual acuity (BCVA) testing, non-contact tonometry, slit lamp biomicroscopy of the anterior eye segment and funduscopy of the posterior eye segment. Digital imaging of the retinal morphology and retinal vessel characteristics of all individuals was acquired with help of color fundus imaging, OCT and OCT-A imaging obtained by DRI Triton Swept-Source OCT-A device (Topcon Corporate, Tokyo, Japan) performed by specially trained technicians. Two ophthalmologists assessed the acquired images regarding artifacts and quality. The quantitative evaluation of the perifoveal retinal vessel density was obtained by analyzing macular OCT-A scans (6 × 6 mm scan field) using the integrated OCT-A analysis software provided by Topcon. The level of segmentation of the different vascular plexuses was predefined by the software. All scans were reviewed and adjusted manually in case of failed segmentation by two ophthalmologists. The OCT-A images were subdivided in central, nasal, temporal, superior and inferior subfields of the retina and the superficial vascular plexuses were measured by the integrated vessel density assessment tool and. Afterwards, central, nasal, temporal, superior and inferior subfields were and analyzed separately regarding retinal vessel density. A mean of the values was calculated for each individual. For further statistical analysis, the results of the retinal vessel densities of both eyes were averaged for each individual as the values were very close in all cases. One control person contributed retinal vessel density data of one eye only due to low quality of the OCT-A image of the contralateral eye even after repeating the imaging.

**Fig. 1:**
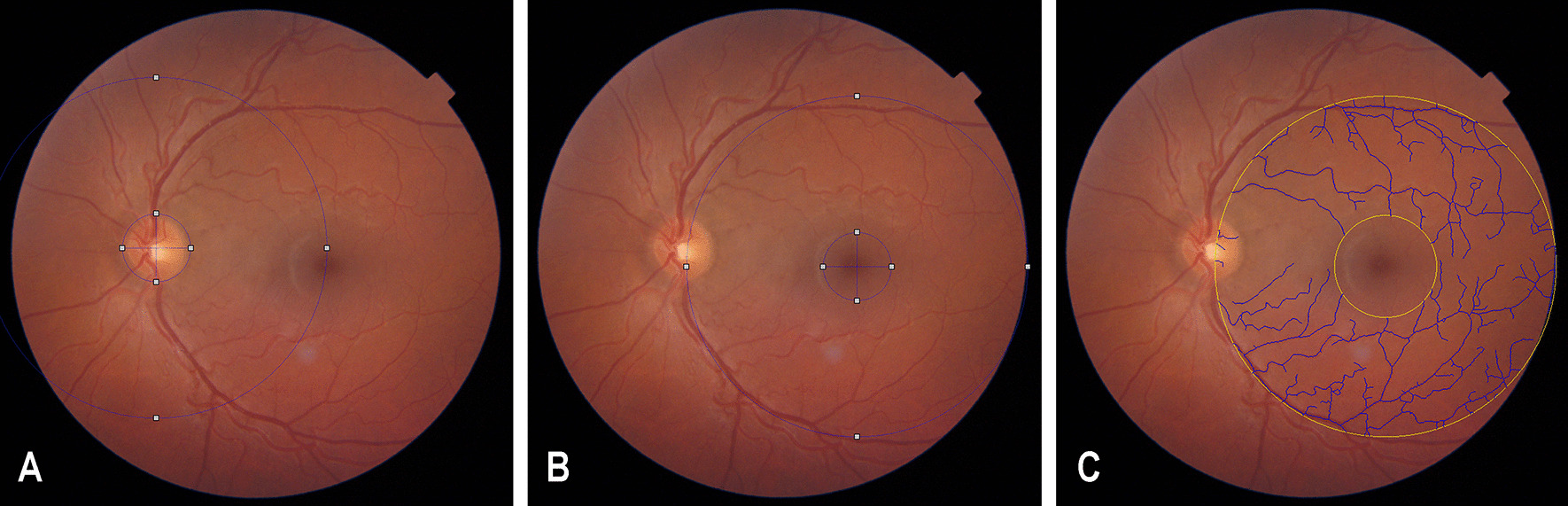
The automatic quantification of the retinal vessel tortuosity index with help of the MONA REVA software is displayed. **A** The optic nerve head and the fovea were marked in the images by an inner and outer circle. **B** The circle was centralized around the fovea. **C** A zone between 1.5 and 5 times the radius of the optic disc, as indicated by the yellow circles, was generated automatically by the software. In this area all vessels > 100 µm were measured automatically

The perimacular retinal vessel tortuosity (further referred to as “retinal vessel tortuosity index”) was measured on the basis of digital color fundus images obtained by the OCT device (Topcon) and automatically quantified using the commercially available MONA REVA software (Version 3.0.0, VITO Health, Mol, Belgium) [24]. First the resolution of the image was determined by choosing “determine from image”. The optic nerve head and the fovea were marked in the images by an inner and outer circle automatically and checked by the observer (Fig. 1A). The circle was centralized perifoveal, as perifoveal vessels, which are predominantly involved in FD [20], should be analyzed (Fig. 1B). A zone between 1.5 and 5 times the radius of the optic disc, as indicated by the yellow circles was automatically generated by the software (Fig. 1C). In this perifoveal area all vessels larger than 100 µm diameter were automatically recognized and measured by the software. The segmentation algorithm is based on a multiscale line filtering algorithm inspired by Nguyen et al. [25]. Post-processing steps such as double thresholding, blob extraction, removal of small-connected regions and filling holes were performed by the software. The automatically calculated retinal vessel tortuosity index represents the mean of the quotients of the end-to-end length and the true length of all vessels within the marked area. For further statistical analysis, the results of the retinal vessel tortuosity indices of both eyes were averaged for each individual as the values were very close in all cases.

### Systemic evaluation

Demographic data including age and gender as well as systemic FD parameters as the genotype and phenotype, MSSI score including general, renal, cardiovascular and neurological subindices and plasma lyso-Gb3 levels were obtained from each patient. Data on ERT or chaperone therapy and other medication (e.g. antihypertensive drugs, anticoagulants) were also collected.

The Mainz Severity Score Index (MSSI) aims to assess the disease severity and is commonly used to monitor disease progression. It represents an established severity scoring system, which evaluates four subdomains (general, cardiovascular, renal, neurological) with 24 items [9, 26, 27].

### Statistical analysis

Statistical analysis was performed with R Core Team (2020, R Foundation for Statistical Computing, Vienna, Austria). When comparing independent groups, the distributions were assessed in regard to outliers, normality and homogeneity if the variances. Outliers were assessed via box plot method. Normality was examined with Shapiro–Wilk test and Q-Q plots. Homogeneity of variances was checked with Levene test. In most cases these assumptions were met and so the independent T-Test was performed. In case of presence of extreme outliers robust Yuen test for trimmed means was performed.

To examine correlation between continuous variables we applied Spearman Rank correlation because of unusual distribution of the residuals. The association between gender and phenotype of FD patients and the retinal vessel tortuosity index was analyzed using the Two-way-ANOVA test. The assumptions for ANOVA regarding normality, homogeneity and absence of outliers were met. A probability of *p* < 0.05 was considered statistically significant in all statistical tests.

## Results

In the presented study, we analyzed different retinovascular parameters of FD patients and their association with systemic parameters of FD. The mean age of the 26 enrolled FD patients was 40.2 years (range 18–56 years) without statistical difference to the control group (mean age 40.2 years; range 21–58 years). All included patients had a genetically confirmed diagnosis of FD, 15 FD patients were female, 11 were male. All individuals with other ocular abnormalities were excluded from the analyses. Seventeen of the 26 participants with FD received ERT at the time point of ophthalmological investigation.

**Fig. 2:**
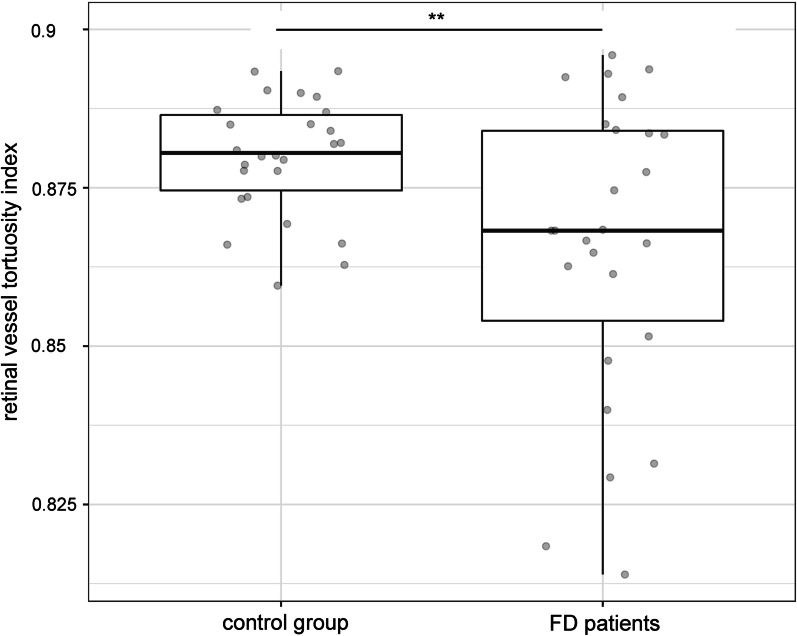
Significantly lower retinal vessel tortuosity indices in FD patients compared to the control group are displayed. **p < 0.01

**Fig. 3:**
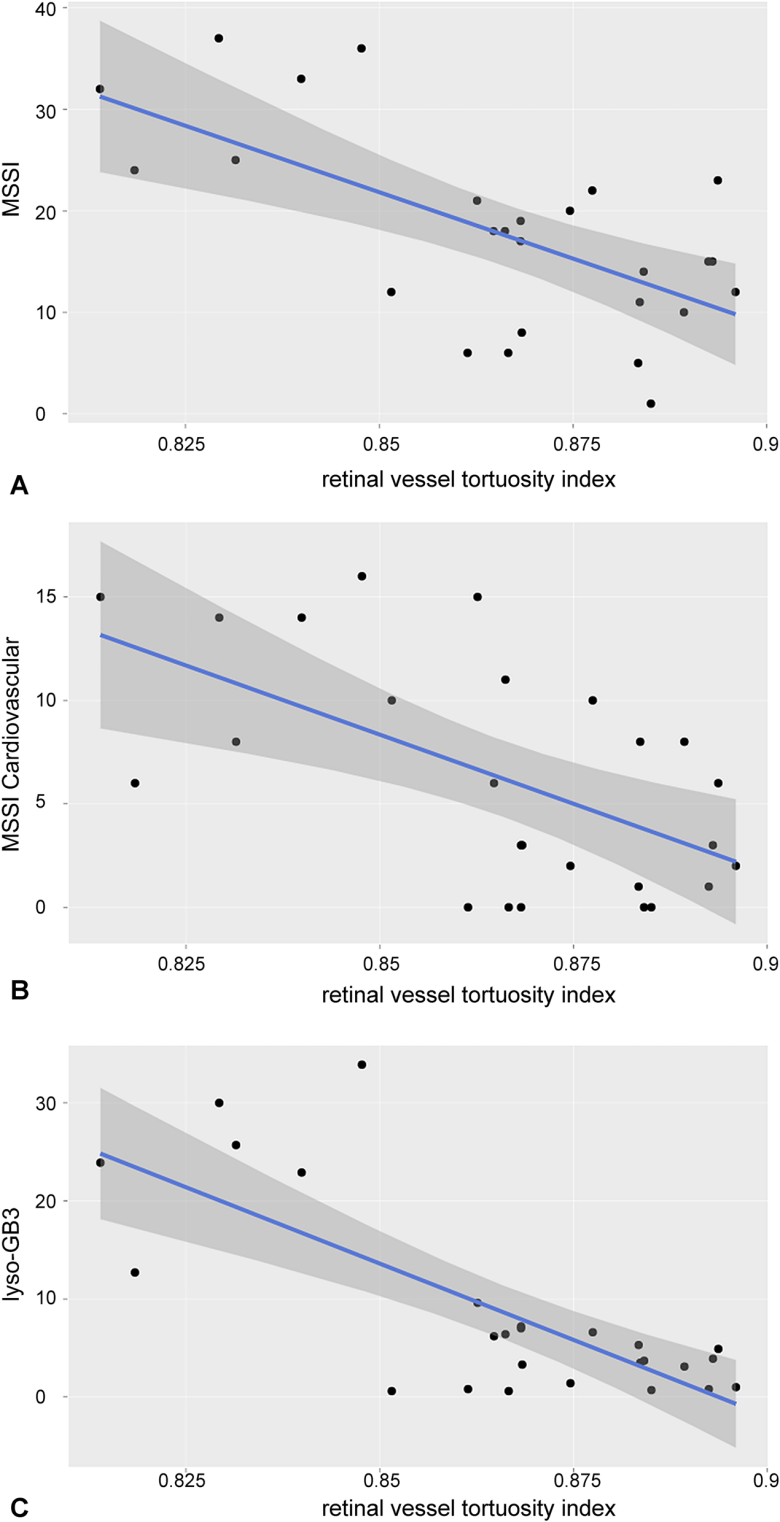
The correlation of retinal vessel tortuosity index of FD patients with systemic parameters is displayed. The retinal vessel tortuosity index correlated significantly negative to the global MSSI (**A** r = − 0.5; *p* < 0.01) and cardiovascular MSSI (**B** r = − 0.5; *p* < 0.01). A significant negative correlation between retinal vessel tortuosity index and the laboratory parameter plasma lyso-Gb3 levels is shown (**C** r = − 0.6; *p* < 0.01)

Investigating the fundus images and the vascular architecture by the MONA REVA software, we detected significantly lower retinal vessel tortuosity indices in FD patients, reflecting a significantly increased vessel tortuosity, compared to the control group (*p* = 0.008; Fig. 2). The retinal vessel tortuosity index of FD patients correlated significantly with the global MSSI (r = − 0.5; *p* < 0.01; Fig. 3A) and cardiovascular MSSI (r = − 0.5; *p* < 0.01; Fig. 3B). In addition, a significant correlation with the laboratory parameter plasma lyso-Gb3 was found (r = − 0.6; *p* < 0.01; Fig. 3C).

FD patients who received ERT (n = 17) had significantly lower retinal vessel tortuosity indices, reflecting a significantly increased vessel tortuosity, compared to untreated patients (n = 9; *p* = 0.02; data not shown). Further subanalyses were performed to understand the impact of the clinical phenotype (classical n = 13 vs. oligosymptomatic form n = 13) and gender on the retinal vessel tortuosity index. FD patients with a classical phenotype (n = 13) showed significantly lower retinal vessel tortuosity indices compared to oligosymptomatic individuals (n = 13; *p* = 0.003).

**Fig. 4:**
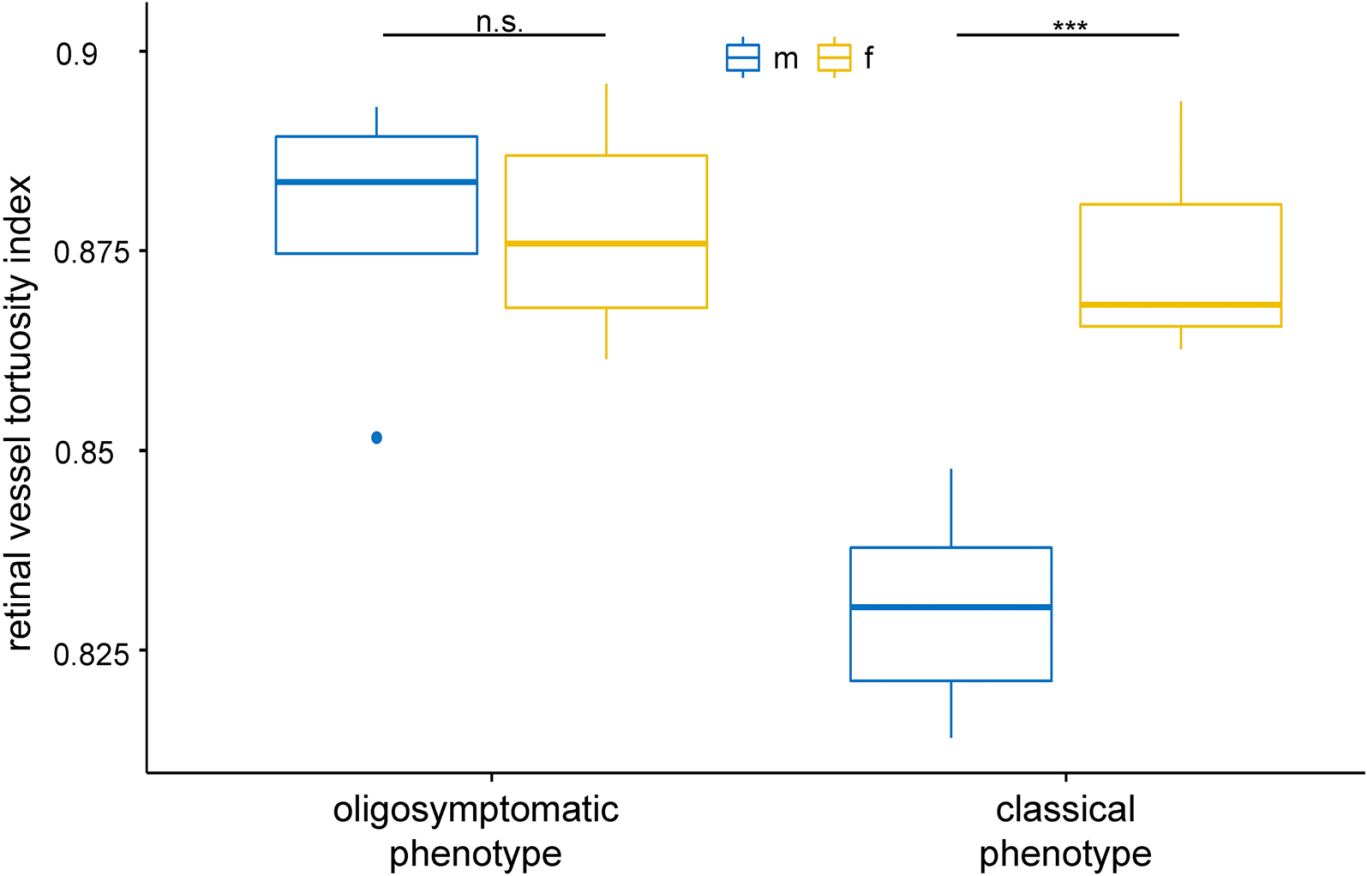
The subanalysis of the impact of the clinical course (classical or oligosymptomatic form) and gender of the FD cohort on the retinal vessel tortuosity index is displayed. Male patients with a classical phenotype (n = 6) show significant lower retinal vessel tortuosity indices, in comparison to females with a classical phenotype (n = 7), males with an oligosymptomatic phenotype (n = 5) or females with an oligosymptomatic phenotype (n = 8). There were no gender specific differences in the retinal vessel tortuosity indices of the oligosymptomatic cohort. Statistical analyses of data were performed with the Two-way-ANOVA test. n.s.: not significant; ***p < 0.001

As shown in Fig. 4, males with classical phenotype (n = 6) show significantly lower retinal vessel tortuosity indices, reflecting a significantly increased vessel tortuosity, in comparison to females with classical phonotype (n = 7), males with oligosymptomatic phenotype (n = 5) or females with oligosymptomatic (n = 8) phenotype (*p* < 0.001). Gender specific differences in the retinal vessel tortuosity indices were missing in the oligosymptomatic cohort.

**Fig. 5:**
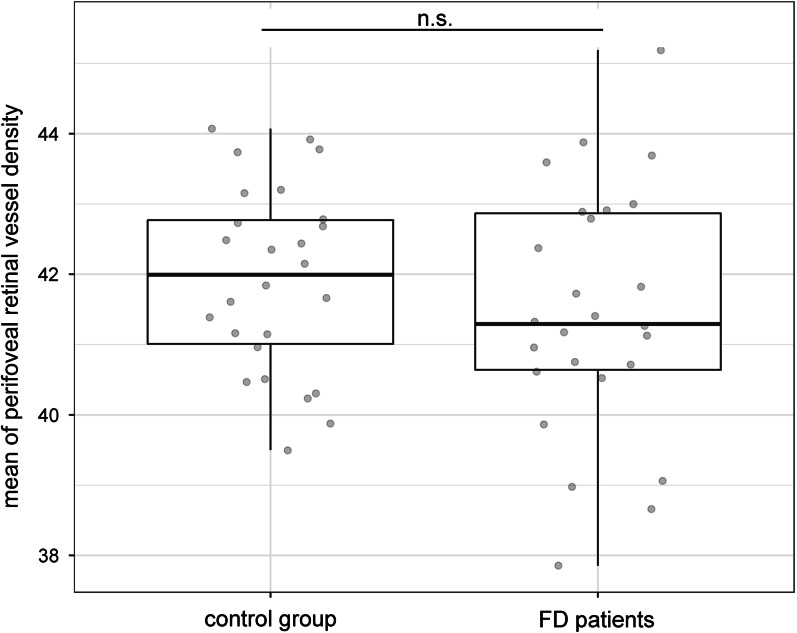
Perifoveal retinal vessel density of the superficial vascular plexus in FD patients compared to the control group are displayed. A broad spectrum of values was detected. n.s.: not significant

Perifoveal retinal vessel density of the superficial vascular plexus was investigated by OCT-A imaging. There were no significant differences between perifoveal retinal vessel density in FD patients and the control persons regarding all analyzed subfields (central, nasal, temporal, superior and inferior subfields) and no differences in the mean value (Fig. 5). Within the FD patient cohort, no statistically significant difference could be detected between patients who were receiving ERT and untreated patients. Furthermore, no correlation between perifoveal retinal vessel density scores and further systemic severity parameters (lyso-Gb3, MSSI and subindices of MSSI) could be shown. There was no association between phenotype and gender of FD patients and perifoveal retinal vessel density values. Furthermore, the age of the patients had neither impact on the retinal vessel tortuosity index nor on the perifoveal retinal vessel density.

## Discussion

Vascular pathology and concomitant organ damage due to storage of Gb3 is assumed to be the a major cause of morbidity and mortality in FD patients [2]. The presented study evaluated two different approaches to characterize the retinovascular status of 26 FD patients and correlated the obtained data to the systemic disease burden.

The eye has ever been an ideal tissue to visualize systemic vascular abnormalities and FD patients are known to have retinal vessel tortuosity. In the data of the Fabry Outcome Survey retinal vessel tortuosity was observed in 21.9% of females and in 48.7% of males. It was more frequently observed in patients with a higher severity score and greater impairment of renal and cardiac function, suggesting a possible association with more severe disease. In the past only the subjectively assessed presence or absence of the retinal vessel tortuosity, without any attempt to grade the severity, was documented, implementing a high subjective bias [13]. Subsequently, a computer-assisted evaluation of retinal vessel tortuosity was presented in FD. Three mathematically calculated parameters of retinal vessel tortuosity showed significant higher values in FD patients compared to healthy individuals. Significantly higher numbers of these parameters were shown in hemizygous males than in heterozygous females. However, a correlation to systemic disease parameters was not performed [18]. San Roman et al. quantified retinal vessel tortuosity semi-automatized in a FD cohort of ten patients with one specific mutation (p.M187R) and found significant correlations between retinal vessel tortuosity and systemic severity measured by the general MSSI, renal MSSI, and neurological MSSI [20]. A further study tested a new semi-automatic software measuring retinal vessel tortuosity from eye fundus images in a group of eleven FD patients and eleven controls. The tortuosity parameters were significantly higher in FD patients in comparison with the controls. No significant association was found between the retinal vessel tortuosity and systemic markers, which may be due to the small number of patients in this study [19]. In our own previous work the investigation of 27 FD patients showed a significant higher retinal vessel tortuosity in FD patients compared to healthy controls as well as a significant correlation with the systemic severity parameter lyso-Gb3 [14]. Taken together, all these previous results indicate that retinal vessel tortuosity represents an important ophthalmological marker in FD. The advantages of a software-assisted analysis of retinal vessel tortuosity are the objectivity, a non-invasive, easily assessable technique and the reproducible comparative manner. This is why we decided to use this method in the current study.

We have shown that automatically quantified retinal vessel tortuosity indices of FD patients were significantly lower, reflecting a significantly increased vessel tortuosity, compared to the control group. Furthermore, males with a classical phenotype show significant lower retinal vessel tortuosity indices in comparison to males with an oligosymptomatic phenotype and females with classical or an oligosymptomatic phenotype. These results match the previously shown data [14, 18–20].

The MSSI as an established measurement tool for the FD severity and progression is used in clinical routine to grade the status of FD patients [9, 26, 27]. Interestingly, the acquired retinal vessel tortuosity index correlated significantly with the systemic disease severity parameter global MSSI and the subindex cardiovascular MSSI, which suggests a potential prognostic role of this ocular clinical parameter. In addition, the significant correlation of the retinal vessel tortuosity index with the plasma lyso-Gb3 levels of the analyzed FD patients supports this hypothesis, because the lyso-Gb3 has been reported to serve as a reliable biomarker for disease progression and therapy efficacy [28, 29]. Therefore, the presented quick, non-invasive, easily assessable, objective and reproducible technique of quantifying the retinal vessel tortuosity software-assisted might contribute to the evaluation of disease severity in FD.

A statistically significant difference or correlation with systemic disease parameters of the quantified perifoveal retinal vessel density, imaged by OCT-A, could not be shown in our study. A previous work, which investigated retinal vessel density with the aid of the OCT-A technique reported increased macular vessel density in the superficial vascular plexus of FD patients compared to healthy controls [21], while another group showed lower macular vessel densities in the superficial and deep vascular plexus of FD patients compared to a control group [22]. In a third, recently published study, the retinal vessel density in the deep vascular plexus was decreased in FD patients compared to controls, without significant difference in the superficial vascular plexus [23].

Influencing factors on vessel density measurements by OCT-A as sex, age, exercise, intraocular pressure, blood pressure [30] or application of mydriatic eye drops [31] even among healthy eyes as well the size of angiocube and interrater reliability [32] were described and may have had an impact on the discrepancies in the different studies. Retinal vessel density, measured by OCT-A, seems to be an inhomogeneous parameter in FD.

## Conclusion

The presented study examined and compared for the first time two different automatically assessed techniques for quantification of retinovascular parameters in FD. Our results favor the acquisition of fundus images and automatically quantifying retinal vessel tortuosity as we could prove a strong correlation between retinal vessel tortuosity indices and the systemic disease severity. The reliability, fast and easily assessable acquisition of fundus images together with a sound scientific background advocates the use of the retinal vessel tortuosity index. This first described, automatized technique represents an investigator-friendly approach with potential use in clinical routine due to its quick and non-invasive manner. Additional prospective longitudinal studies will be necessary in order to evaluate the predictive value of the retinal vessel tortuosity index as well as the potential value on therapeutic decision making.

## Data Availability

The datasets used and/or analysed during the current study are available from the corresponding author on reasonable request.
